# Supplementary material for: Learning curve and surgical outcome of robotic assisted colorectal surgery with ERAS program
Source: Sci Rep. 2022 Nov 29;12:20566. doi: 10.1038/s41598-022-24665-w (PMC9709162; doi:10.1038/s41598-022-24665-w)
Supplement: Supplementary file 1 — Supplementary Information 1. [file 41598_2022_24665_MOESM1_ESM.zip › LSC Raw data-Ñ[▒K/126/H_ñw╝╨░O▒Kñσ.pdf]

正式摘要(電子簽章：000000217257962)

W55 / W55 ☐ 英文標題(注意:無英文姓名-請補病人基本檔!) ☐【讀取】憑證

臺中榮民總醫院

出院病歷摘要

轉入號： 轉出號：

[redacted]

(8)流水編號	
(9)入院日期	110/05/05 (2021) 直腸外科 W55 - 235
(10)轉科日期	
(11)出院日期	110/05/19 (2021) 住院天數計 14 日

診斷

- 入院 :1. Cancer of R-S colon, 15cm FAV, with obstruction and invasion of bladder, intestine and appendix
  PH:
  1. DM 19 Years
  2. HT 2-3 months
  3. BPH, s/p TURP
- 出院 :1. Cancer of R-S colon, 15cm FAV, with obstruction and invasion of bladder, intestine and appendix s/p anterior resection + segmental resection of intestein + appendectomy + bladder wall excision on 2021/05/06
  PH:
  1. DM 19 Years
  2. HT 2-3 months
  3. BPH, s/p TURP

主訴

- colon cancer admitted for Laparoscopic Anterior resection and anastomosis.

病史

- The 53-year-old male had underlying disease of
  1.DM 19 Years
  2.HTN 2-3 months
  3.BPH, s/p TURP
  He suffered from abdominal fullness for months with intermittent bloody stool, and body weight dropped 5-6 kg in 6 months. Stool occult blood showed positive and he underwent colonscope at 竹山秀傳, which showed CA OF RS COLON 15CM FAV, PENDING OBSTRUCTION, PROVED. Therefore, he was admitted for Laparoscopic Anterior resection and anastomosis.

體檢發現

- Anal inspection:
  Recto digital examination:
  Sigmoidoscopy:
  Anoscopy:
  Abdomen:
  - General appearance :
    - easy looking , conscious clear , without cardiopulmonary distress
    - Vital signs自動帶入date 體溫(BT) 脈搏(P) 呼吸(R) 血壓(BP) 身高(BH) 體重(BW) BMI
      20210505 36.3℃ 96次/分 17次/分 140/85mmHg 163.1cm 62.6kg 23.5
    - Vital signs: B.P. 136/94 mmHg , P.R. 108 /min , R.R. 18 /min , B.T. 37 ℃
  - HEENT :
    - Eyes: no anemic conjunctiva, no icteric sclera, normal EOM
    - ENT: no otorrhea, no rhinorrhea, no erythematous mucosa, no injected tonsil, no deformity
  - Neck: supple, freely movable, trachea midline,no deformity, no engorged jugular vein , no palpable thyroid or lymph node enlargement
  - Chest:
    - Inspection: Grossly normal, symmetric and fully expansion, no accessory muscle used, no operation scar, no spider angiomata, no gynecomastia
    - Palpation: no palpable mass or nodule, no subcutaneous emphysema, no tenderness
    - Ausculation: clear breathing sound
  - Heart:
    - Percussion: normal heart size
    - Ausculation: regular heart beat, normal S1, S2, no gallop, no murmur
  - Abdomen:
    - Inspection: in
      - flat contour, symetrically, no scar, no stria, no dilated veins, no rashes
    - Ausculation: normoactive bowel sound, no abdominal bruits
    - palpation: soft, no muscle guarding or rigidity, no local tenderness, no rebounding pain, no palpable mass, no palpable pulsation
    - Percussion: no shifting dulllness, no hepatomegaly, no splenomagaly
  -

Peripheral pulsation :	CA	BA	RA	FA	PA	PTA	DPA
Right side :	++	++	++	++	++	++	++/+++
Left side :	++	++	++	++	++	++	++/+++

  - Genito-anus: no tenderness or palpable mass lesion or rectal shelf, normal external genitalia, normal anal tone, brown stool

CRS CONDITION

  -
    - Lung

手術日期及方法

- 手術日期及方法20210506 1. Radical laparoscopic anterior resection with end-to-end anastomosis (conversion to open), ILS 29mm, bilateral doughnuts: intact, re-inforcment with silk: nil. Air leak test: done, no air leak.
  2. High ligation (-) below left colic artery.
  3. Cut margin: proximal 10cm, distal 6cm.
  4. Take down of splenic flexure: nil.
  5. Reperitonealization: nil.
  6. One 12mm penrose drain at pelvis.
  7. Segmental resection with side to side anastomosis with lineal stapler
  8. Appendectomy was done
  9. Partial cystectomy due to tumor involvement
  20210506 Bladder, repair

住院治療經過

- After admission, anterior resection + segmental resection of intestein + appendectomy + bladder wall excision was done smoothly on 2021/05/06 under no contraindication. Under stable condition, he was discharge and arranged OPD followed up.

合併症

一般檢查

- 累積報告 :

DATE	TIME	ALB	TP	BIL.T	BIL.D	ALKP	AST	ALT	LDH	CHOL	TG	HDL-C	LDL	UA	NH3	AMY
1100506	1147		7.6							204	98	42	138	3.9		
1100507	0851	3.0		0.47	0.12	74	12	11								

1100511	0852	3.1		0.29			8	7								

DATE	TIME	WBC	RBC	HGB	HCT	MCV	PLT	BLST	NEUT	BAND	SEG	LYM	MON	EOS	BAS
1100507	0903	8860		10.0			428		74.4			19.9	4.9	0.5	0.3
1100511	0858	9370	3.80	10.5			511		73.7			14.3	5.5	6.1	0.4

DATE	TIME	NA	K	CL	CA	BUN	CREAT	Mg	P	CRP	ESR	PCT	LIPASE	LACTATE
1100506	1147									2.679				
1100507	0810													22.5
1100507	0851	137	4.3		7.9	13	0.72							
1100511	0852	136	4.0	102	8.6	11	0.64							

DATE	TIME	GLUCOSE	PAB	TRF	ZN	CU	KETONE	CK	CKMB	PT-P	PT-C	APTT-P	APTT-C	TRO-T
1100507	0906	210												

DATE	TIME	PH	BEB	SCVO2	PO2	HCO3	HGB	PCO2	TCO2
1100507	0816	7.422	-0.2	97.3	92.3	23.9		37.5	25.1

- 放射線報告 :檢驗項目 **:CT ABDOMEN-SURVEY** 報告日期 **:1100511**

  主 訴：
  Result:Y
  Finding:
  Imaging Report Form for Colorectal Carcinoma (AJCC 8th)
  1. Imaging Date: 20210506
  Imaging Modality, Imaging by
  CT scan
  Imaging Protocol: CT scan with oral contrast medium and post-IV contrast enhancement
  2. Tumor location / Size
  Location:
  Sigmoid
  Size:
  Measurable: about 8 cm (largest diameter)
  3. Tumor invasion
  Tumor invades non-peritonealized pericolonic or perirectal tissues
  4. Regional nodal metastasis
  Not assessable
  5. Distant metastasis (In this study)
  No or Equivocal
  6. Other findings: > Segmental irregular wall thickening over S-colon, with some areas of pericolonic fat stranding appearance, with or without involvement of urinary bladder. Colon cancer could be compatible. Suggest clinical correlation and further evaluation to R/O other possibility.
  > Several clustered and/or relatively prominent nodules/lymph nodes around the lesion, metastatic lymphadenopathies can not be completely ruled out. Please correlate with clinical condition.
  > R/O small gall bladder stones
  > Presence of fat herniation over bilateral inguinal regions.
  Impression:
  Please see descriptions.

特殊檢查

病理報告

其它

出院時情況

- 3.改門診治療

出院指示

出院計劃

主治醫師 2062F 蔣鋒帆 / 住院醫師 9760F 趙[illegible]

正式摘要(電子簽章：**000000217257962)**

W55 / W55

doctorRank:B
